# Rapid and Efficient Synthesis of Succinated Thiol Compounds via Maleic Anhydride Derivatization

**DOI:** 10.3390/molecules30030571

**Published:** 2025-01-27

**Authors:** Hiroshi Yamaguchi, Hikari Sugawa, Himeno Takahashi, Ryoji Nagai

**Affiliations:** 1 Department of Food and Life Science, School of Agriculture, Tokai University, 871-12 Sugido, Mashiki, Kamimashiki, Kumamoto 861-2205, Japan; sugawa.hikari.h@tokai.ac.jp (H.S.); nagai-883@umin.ac.jp (R.N.); 2 Graduate School of Bioscience, Tokai University, 871-12 Sugido, Mashiki, Kamimashiki, Kumamoto 861-2205, Japan; 4ctld001@tokai.ac.jp

**Keywords:** succination, maleic anhydride, thiol compound, Michael addition reaction

## Abstract

Succination is a non-enzymatic post-translational modification of cysteine (Cys) residues, resulting in the formation of *S*-(2-succino)cysteine (2SC). While hundreds of 2SC-modified proteins have been identified and are associated with the dysfunction of proteins, the underlying molecular mechanisms remain poorly understood. Conventional methods for synthesizing succinated compounds, such as 2SC, often require prolonged reaction times and/or HCl hydrolysis. In this study, we present a rapid and efficient synthesis method for succinated compounds using maleic anhydride, enabling more effective in vivo studies of succination mechanisms. This method was tested on thiol compounds with varying molecular weights, including Cys derivatives, Cys-containing peptides, and reduced bovine serum albumin. By incubating these compounds in an aqueous buffer with maleic anhydride dissolved in an organic solvent like diethyl ether, we achieved significantly improved succination efficiency compared to conventional methods. The succination efficiency using maleic anhydride surpassed that of fumaric acid or maleic acid. Notably, this approach facilitated the succination of amino acids, peptides, and proteins within minutes at 25 °C, without requiring acid hydrolysis. Our findings provide a straightforward, time-efficient strategy for synthesizing succinated thiol compounds, offering a valuable tool to enhance the understanding of succination’s molecular mechanisms and its role in protein function and dysfunction.

## 1. Introduction

Post-translational modifications (PTMs) of proteins are critical for regulating their function and facilitating signal transduction. Among these, modifications of the thiol group in cysteine (Cys) residues are particularly important due to the thiol group’s high nucleophilicity. One prominent PTM involving Cys residues is the formation of disulfide bonds, which are covalent cross-links between the side chains of two Cys residues. These bonds play a crucial role in stabilizing protein conformation. Additionally, the redox modulation of Cys residues is essential for various biological processes, including transcription, protein expression, and signal transduction [1,2,3,4]. Aberrations in these modifications can contribute to the development of disease pathologies.

Succination is a non-enzymatic PTM that involves the Michael addition of a thiol group to an α,β-unsaturated carboxyl compound, producing *S*-(2-succino)cysteine (2SC) residues. This modification is irreversible under physiological conditions [5]. Mutations in fumarate hydratase, an enzyme in the tricarboxylic acid (TCA) cycle, lead to fumaric acid accumulation at millimolar levels [6]. Excess fumaric acid can react with the thiol groups of Cys residues, resulting in 2SC formation [7,8]. Hundreds of succinated proteins have been identified in vivo [9], including key proteins with functional Cys residues, such as glyceraldehyde-3-phosphate dehydrogenase [10], adiponectin [11], and tubulin [12]. These 2SC modifications can impair protein functionality, with the extent of succination directly correlating to cellular fumaric acid levels. Consequently, 2SC is considered a valuable biomarker for conditions associated with fumaric acid accumulation and heightened succination, such as hyperglycemia linked to type 2 diabetes and obesity [13].

Thiol-reactive compounds are categorized based on their reactivity and include alkylation reagents, double-bond addition reagents, disulfide-forming agents, and sulfenylation reagents [14,15]. *N*-Ethylmaleimide and its derivatives, known for their reactivity with thiol groups, exhibit high specificity and stability in aqueous solutions, making them widely used in protein and peptide chemistry [14,15,16]. Existing methods for 2SC synthesis include reacting Cys with fumaric acid in buffered solutions over several days [5,17]. However, prolonged reactions may trigger undesirable oxidation reactions, leading to side reactions such as the formation of disulfide bonds. Another approach involves acidic hydrolysis of *N*-ethylmaleimide-modified Cys [7]; however, the high temperature and 6 M HCl conditions required are unsuitable for synthesizing 2SC-modified peptides or proteins, as these conditions also cleave peptide bonds. Thus, there is a pressing need for a rapid, mild, selective, and efficient synthesis method for succinated compounds to advance our understanding of succination’s molecular mechanisms in vivo.

In this study, we demonstrate that maleic anhydride serves as an effective reagent for the synthesis of succinated compounds. Under optimized reaction conditions, we achieved efficient synthesis of 2SC amino acids at room temperature in a short timeframe, without the need for acidic or harsh reagents. Furthermore, we extend this method to synthesize 2SC-modified protein and peptide.

## 2. Results and Discussion

### 2.1. Synthesis Strategy of Succinated Compounds

**Figure 1:**
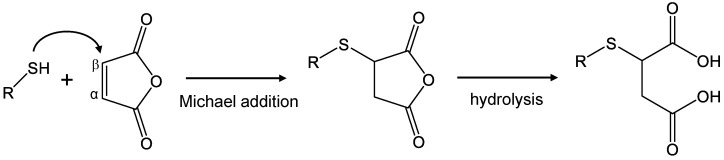
Schematic representation of the succination reaction. When the nucleophilic thiol group attacks the electrophilic β-carbon atom of maleic anhydride, a thioether bond is formed through Michael addition. Subsequently, the anhydride portion undergoes hydrolysis, and a succinated compound is formed.

The double bond of maleimide is highly reactive toward nucleophilic thiol groups through Michael addition, facilitated by the electron-withdrawing effect of the two adjacent carbonyl groups [14]. This property makes chemical derivatization of thiols with maleimides an effective strategy for preparing various bioconjugated samples [14,15]. Although *N*-ethylmaleimide-modified Cys can serve as a precursor for 2SC synthesis, the ring-opening of its five-membered ring necessitates acidic hydrolysis [7]. In vivo, succination is believed to occur when fumaric acid, a product of the TCA cycle, reacts with thiol groups [7,17]. However, our results showed that when acetyl-cysteine (Ac-Cys) was mixed with fumaric acid in a neutral buffer, succination progressed only minimally even after 30 h of reaction, with the main by-product being a dimer of Ac-Cys formed through a side reaction (Figure S1a). This suggests that in vivo succination depends on factors such as reactant concentrations, pH, and redox state. Despite this, the precise mechanism of succination remains unclear. One potential limitation is the steric hindrance around the double bond in fumaric acid, which likely impedes nucleophilic reactions with thiols. To address this, we explored the reaction between thiol compounds and maleic acid, a geometric isomer of fumaric acid. After a 30 min reaction, succination occurred in less than 10% of the starting material, and the reaction efficiency were insufficient. After a 30 h reaction, the peak of the starting material disappeared, and Ac-2SC was detected as the main product, with a dimer of Ac-Cys detected as a by-product (Figure S1b). These results suggest that rapid succination is important for avoiding side reactions and preventing a decrease in reaction efficiency. Therefore, we evaluated maleic anhydride, which, like maleimide, is more reactive toward thiols due to the lower energy level of its lowest unoccupied molecular orbital (LUMO). Maleic anhydride decomposes rapidly into maleic acid in aqueous solutions, necessitating its dissolution in an organic solvent to preserve its reactivity. Conversely, thiol compounds, including Cys derivatives, Cys-containing peptides, and proteins with free thiol groups, are best dissolved in aqueous solutions to maintain their solubility and structural integrity. Based on these considerations, we devised a strategy to react thiol compounds dissolved in an aqueous buffer with maleic anhydride dissolved in an organic solvent (Figure 1).

### 2.2. Succination Using Maleic Anhydride

To evaluate our synthetic strategy, we examined several reactants and solvents for their ability to facilitate succination. Acetyl-cysteine (Ac-Cys) was selected as a model thiol compound. Initially, the effect of the solvent on maleic anhydride derivatization was investigated using diethyl ether, ethyl acetate, and water. Diethyl ether and ethyl acetate, with water solubilities of 6.1 g/100 mL and 8.3 g/100 mL, respectively, were chosen with the expectation that maleic anhydride dissolved in these organic solvents would gradually react with thiol compounds in the aqueous buffer solution. The reactions were monitored using HPLC, and the resulting compound structures were analyzed via MS. When the thiol group reacts with the double bond of maleic anhydride, akin to its reaction with maleimide, a thioether bond forms, increasing the molecular weight by 116 (succination). Conversely, if the thiol group reacts with the acid anhydride bond, forming a thioester bond, the molecular weight increases by 98 (maleylation).

**Figure 2:**
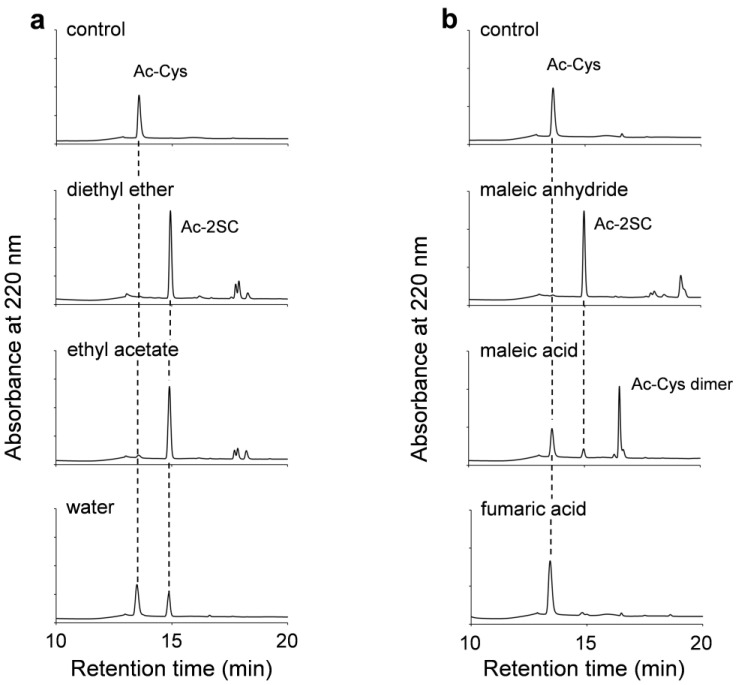
Succination of Ac-Cys. (**a**) HPLC profile of Ac-Cys and its succinated product (Ac-2SC) with maleic anhydride in different solvents: control (no reaction); diethyl ether; ethyl acetate; water. (**b**) HPLC profile of the succination reaction using maleic anhydride, maleic acid, or fumaric acid dissolved in diethyl ether. All succination reactions were carried out at 25 °C for 30 min in 100 mM phosphate buffer (pH 7.0). The concentrations of Ac-Cys and carboxyl compounds were 0.2 μmol and 4 μmol, respectively.

As shown in Figure 2a, when diethyl ether or ethyl acetate was used as the solvent, the HPLC peak corresponding to Ac-Cys completely disappeared, and a new peak with a retention time distinct from Ac-Cys was observed. MS analysis confirmed that this new compound had a molecular weight increase of 116.0213 relative to Ac-Cys ([M + H]^+^ = 122.0275), corresponding to Ac-2SC ([M + H]^+^ = 238.0488). These findings indicate that the reaction between maleic anhydride and thiols proceeds through Michael addition, forming a thioether bond, with succination occurring via ring opening in the presence of water. We also analyzed three peaks around a retention time of 18 min. The results showed that the peaks corresponded to [M + H]^+^ = 425.0685, 425.0689, and 425.0683, which matched the theoretical values of by-products formed when the thiol group of Ac-Cys undergoes a nucleophilic reaction on the carbonyl group during the ring opening step after the Michael addition reaction with maleic anhydride (Figure S2). Furthermore, since all three peaks had the same *m*/*z* value, it is suggested that the nucleophilic reaction of Ac-Cys may have led to the formation of structural isomers or enantiomers. In contrast, when water was used as the solvent, 79% of Ac-Cys remained unreacted, although Ac-2SC was detected. These results suggest that the rapid decomposition of maleic anhydride in an aqueous environment is mitigated when dissolved in an organic solvent, as anticipated. Considering the ease of solvent removal from the reaction vessel, diethyl ether was selected for the maleic anhydride reaction in subsequent experiments. The reactions were performed at 25 °C due to diethyl ether’s low boiling point of approximately 35 °C.

Additionally, we investigated whether Ac-2SC could be synthesized using fumaric acid or maleic acid dissolved in diethyl ether. As shown in Figure 2b, succination comparable to that achieved with maleic anhydride was not observed. Instead, a dimer of Ac-Cys was detected as a by-product in the reaction with maleic acid. These results confirm that maleic anhydride exhibits higher reactivity and lower steric hindrance compared to fumaric acid and maleic acid, validating its suitability for efficient succination.

### 2.3. Optimization and Characterization of Succination Using Maleic Anhydride

**Figure 3:**
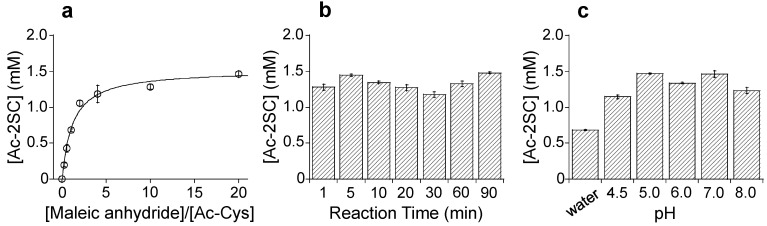
Characterization of succination using maleic anhydride. (**a**) Influence of the Ac-Cys to maleic anhydride ratio in 100 mM phosphate buffer (pH 7.0) at 25 °C for 30 min. (**b**) Time course of the succination of Ac-Cys in 100 mM phosphate buffer (pH 7.0) at 25 °C. (**c**) Effect of pH on the succination of Ac-Cys. The reaction was conducted at 25 °C for 30 min. In all experiments, maleic anhydride was dissolved in diethyl ether and used in the succination reaction. The concentrations of Ac-Cys and maleic anhydride were 0.2 μmol and 4 μmol, respectively. The graphs show the mean ± standard deviation of at least three experiments.

To achieve efficient synthesis of succinated thiol compounds, the derivatization reaction was optimized. First, the influence of the molar ratio of Ac-Cys to maleic anhydride was evaluated. HPLC analysis revealed that sufficient succination occurred with approximately a 10-fold molar excess of maleic anhydride (Figure 3a). For a complete reaction, a 20-fold excess was selected as the optimized condition. Next, the reaction efficiency over varying time intervals was assessed. As shown in Figure 3b, 64% of Ac-2SC was synthesized within 1 min at 25 °C in 100 mM phosphate buffer (PB; pH 7.0). Extending the reaction time to 90 min resulted in a marginal increase, with a yield of 74%, indicating that the reaction rapidly reaches a near-maximum efficiency.

Since maleimides are known to selectively react with thiol groups in aqueous solutions at pH 6.5–7.5 to form stable thioether bonds [16], the effect of pH on the succination reaction was examined (Figure 3c). Maleic anhydride reacted with thiol groups effectively across a broad pH range, with reaction efficiencies of ≥70% observed at neutral pH. In aqueous solutions, hydrolysis of maleic anhydride to maleic acid caused acidification, which likely reduced reaction efficiency under lower pH conditions. These results suggest that the reaction between maleic anhydride and thiol groups follows the Michael addition mechanism, similar to maleimide-thiol reactions, and is most effective under mild conditions (25 °C and neutral pH).

To investigate the reactivity of maleic anhydride with other amino acid side chains, succination experiments were conducted using several model peptides under optimized conditions (25 °C, 30 min, pH 7.0 buffer, and a 20-fold excess of maleic anhydride relative to peptide concentration). The observed masses for each peptide are summarized in Table 1. Peptide 1, with an acetyl-protected *N*-terminal amino group, showed a mass increase of 116 units due to succination. In contrast, peptide 2, which lacked *N*-terminal protection, exhibited an increase of 214 mass units (116 from succination + 98 from maleylation of the *N*-terminal amino group). Similar maleylation patterns were observed for peptides 3 and 4. For peptide 5, a mass increase of 196 units (2 × 98) indicated maleylation at both the *N*-terminal amino group and the ε-amino group on the Lys side chain. The data from the five model peptides confirmed that maleic anhydride does not react with acidic side chains (Asp), basic side chains (Arg and His), neutral side chains (Asn, Ser, Thr, and Tyr), or polar side chains (Met, Trp, and biotin). These results align with a previous study by Tian et al. [18], which demonstrated that maleic anhydride reacts with *N*-terminal and ε-amino groups in Lys side chains, resulting in maleylation. Based on these findings, it was concluded that protecting amino groups is necessary when synthesizing succinated peptides, depending on peptide structure. However, protection of other functional groups is generally unnecessary.

Additionally, unmodified peptides were detected at levels of 10–30% in reactions where maleylation occurred, as shown by HPLC analysis (profiles not displayed). In Tian et al.’s study, complete maleylation of peptides at room temperature in 100 mM NaHCO_3_ required a 100-fold molar excess of maleic anhydride in methanol and at least 1 h of reaction time [18]. This indicates that maleic anhydride exhibits higher reactivity toward thiol groups than amino groups. The pK_a_ values of thiol groups in Cys (8.18) and ε-amino groups in Lys (10.53) suggest that, under neutral conditions, thiol groups possess greater nucleophilicity toward the conjugated double bond of maleic anhydride, supporting their preferential reactivity.

### 2.4. Scale-Up of Succinated Compound Synthesis

To evaluate the practicality of our synthetic method for producing succinated compounds, we scaled up the reaction system. Ac-2SC synthesis was conducted at scales of 10, 20, and 50 µmol by reacting Ac-Cys in 1 mL of 200 mM PB (pH 7.0) with maleic anhydride dissolved in 4 mL of diethyl ether. Maleic anhydride was added in a 20-fold molar excess relative to the Ac-Cys concentration. The reaction was carried out at 25 °C for 30 min. Ac-2SC was successfully synthesized with yields of 5.4 ± 0.2, 13.4 ± 0.5, and 35.4 ± 0.1 µmol for the 10, 20, and 50 µmol scales, respectively. The crude product from the 50 µmol scale was purified using HPLC, resulting in 9.6 mg of Ac-2SC with a purity > 98% and a yield of 69%. These results demonstrate that the proposed method is efficient and practical for compound synthesis at the laboratory scale.

### 2.5. 2SC Synthesis

We further explored the synthesis of 2SC at the laboratory scale using the developed method. As maleic anhydride can react with the *N*-terminal amino group of peptides, the use of unprotected *N*-terminal Cys as the starting material led to the synthesis of compounds exhibiting both succination and maleylation, as anticipated (Figure S3). To prevent *N*-terminal maleylation, Boc-Cys was selected as the starting material. The synthetic strategy involved two steps: first, Boc-Cys was reacted with maleic anhydride to produce Boc-2SC. Following purification, the Boc protecting group was removed using a 90% TFA aqueous solution, yielding 2SC. This deprotection reaction generates *tert*-butanol and CO_2_ as by-products, which are easily eliminated by freeze-drying, simplifying the process and minimizing yield loss during purification.

**Figure 4:**
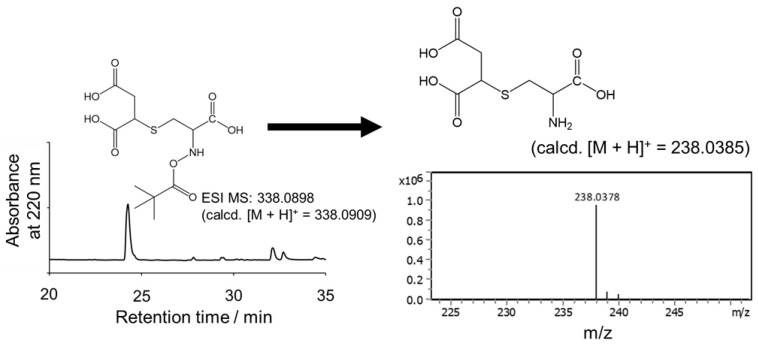
2SC synthesis. The succination of Boc-Cys was carried out at 25 °C for 30 min in 200 mM phosphate buffer (pH 7.0). Maleic anhydride, dissolved in diethyl ether, was added in a 20-fold excess. The de-Boc reaction was performed in a 90% TFA aqueous solution.

The synthesis of 2SC was performed at a 20 µmol scale by reacting Boc-Cys in 1 mL of 200 mM PB (pH 7.0) with maleic anhydride added in a 20-fold molar excess. The re-action was carried out at 25 °C for 30 min. As shown in Figure 4, Boc-2SC was synthesized with high purity. The crude product was purified via HPLC, lyophilized, and subsequently treated with 90% TFA aqueous solution, resulting in a 77% yield (3.6 mg) of 2SC. MS analysis confirmed the structural integrity of 2SC following TFA treatment. Although previous 2SC syntheses using HCl hydrolysis of N-ethylmaleimide-modified Cys [7] do not provide yield data, a direct comparison of reaction efficiency was not possible. Nevertheless, the yield achieved in this study is considered sufficient for practical applications at the laboratory scale.

### 2.6. Succinated BSA Synthesis

The succination of Cys residues in proteins using maleic anhydride was investigated, with BSA serving as a model protein. BSA contains 17 disulfide bonds and one reduced Cys residue, and upon reduction, it possesses 35 free thiol groups. Common protein reducing agents, such as dithiothreitol or 2-mercaptoethanol, are thiol-based and prone to side reactions with maleic anhydride. To avoid such complications, TCEP, a non-thiol reducing agent, was employed. The synthesis strategy began with reducing BSA using TCEP to cleave disulfide bonds. Maleic anhydride, dissolved in diethyl ether, was then added to the reaction mixture to carry out succination. However, preliminary experiments revealed that reduced BSA formed aggregates, rendering it unsuitable for further reactions. This aggregation was attributed to undesirable conformational changes following disulfide bond cleavage. To preserve the protein’s solubility post-reduction, a denaturant was incorporated into the reduction process. While urea and guanidine hydrochloride are commonly used protein denaturants, urea was avoided due to its potential reactivity with maleic anhydride. Guanidine hydrochloride was selected instead, as its guanidino group, present in Arg side chains, does not react with maleic anhydride (see Table 1).

The succination reaction was performed at 25 °C for 1 h. Post-reaction, excess maleic anhydride, its hydrolysis products, TCEP, and guanidine hydrochloride were removed via dialysis. Starting with 30 μM (1.98 mg/mL) BSA, 22.4 μM (1.48 mg/mL) succinated BSA was recovered after dialysis, yielding 75%. The modified BSA was evaluated via ELISA to assess recognition by an anti-2SC monoclonal antibody [19]. For comparison, BSA modified by reacting with maleic acid in aqueous solution over 2 days was also tested. ELISA results indicated that, at equivalent antigen concentrations, BSA modified with maleic anhydride was recognized as strongly as BSA modified with maleic acid (Figure S4). These findings confirm that succination using maleic anhydride achieves higher reaction efficiency, even in the presence of protein thiol compounds.

## 3. Materials and Methods

### 3.1. Materials

Ac-Cys and 9-fluorenylmethoxycarbonyl (Fmoc-) amino acids were obtained from Sigma-Aldrich (St. Louis, MO, USA). Diethyl ether, *N*,*N*-diisopropylethylamine (DIEA), *N*-methylpyrrolidone (NMP), piperidine, and trifluoroacetic acid (TFA) were sourced from Fuji Film Wako Pure Chemical (Osaka, Japan). BSA and tris(2-carboxyethyl)phosphine hydrochloride (TCEP) were purchased from Nacalai Tesque (Kyoto, Japan). Maleic anhydride, maleic acid, fumaric acid, and *tert*-butoxycarbonyl-cysteine (Boc-Cys) were supplied by Tokyo Chemical Industry (Tokyo, Japan). HBTU and HOBt were procured from Peptide Institute (Osaka, Japan). All other reagents used were of analytical grade.

### 3.2. Succination

Succination reactions were performed by adding an excess of maleic anhydride dissolved in diethyl ether to thiol compounds, including Ac-Cys, Cys, Boc-Cys, and Cys-containing peptides, dissolved in buffer. The maleic anhydride solution in diethyl ether was freshly prepared before each use. For optimization of reaction conditions, 0.4 mL of the diethyl ether solution containing maleic anhydride was added to 0.1 mL of a 100 mM buffer solution containing 2 mM of thiol compound. The reaction was conducted at 25 °C and terminated by the addition of acetic acid to achieve a final concentration of 10%. The effects of pH (4.5–8.0) and reaction time (1–90 min) on succination were evaluated. After separating the diethyl ether phase, the reaction efficiency was assessed by quantifying thiol and succinated compounds in the buffer solution using high-performance liquid chromatography (HPLC). HPLC analysis was performed using a UV-2075 Plus Intelligent UV–Vis detector and a PU-2089i Plus pump (Jasco, Tokyo, Japan) at 30 °C. The elution process utilized solution A (H_2_O/0.05% TFA) and solution B (CH_3_CN/0.04% TFA). The column used was a Cosmosil 5C18-AR-II (4.6 × 250 mm). A linear gradient of 0–60% solution B over 30 min was applied at a flow rate of 1.0 mL/min. The molecular weights of the target compounds were confirmed using liquid chromatography quadrupole time-of-flight mass spectrometry (LC-QTOF-MS) (Bruker Daltonics, Billerica, MA, USA). Lyophilized samples were reconstituted in 50% CH_3_CN. The mobile phase consisted of solution A (H_2_O/0.1% formic acid) and solution B (CH_3_CN/0.1% formic acid), and measurement was performed in the isocratic mode with 50% solvent B. The flow rate was set to 0.2 mL/min, and the injection volume was 3 μL. All measurements were conducted in positive mode and ionization source temperature was 200 °C. Data were acquired with a stored mass range from 50 to 1500 (*m*/*z*).

### 3.3. 2SC Synthesis

Four milliliters of a 100 mM maleic anhydride solution in diethyl ether were added to 1 mL of a 20 mM Boc-Cys solution prepared in 200 mM phosphate buffer (PB) at pH 7.0. The reaction was conducted at 25 °C for 30 min in a 15 mL polypropylene centrifuge tube to synthesize Boc-2SC. The Boc-2SC product was purified using HPLC with a C18 column (9.4 × 250 mm) and a mobile phase of 0.05% TFA in H_2_O/CH_3_CN. The purified product was lyophilized and resuspended in a 90% TFA aqueous solution for Boc group deprotection, which was carried out at room temperature for 30 min. After the reaction, the solution was lyophilized, yielding 2SC without further purification.

### 3.4. Succinated BSA Synthesis

BSA (30 μM) was reduced with 5 mM TCEP in 200 mM PB (pH 7.0) containing 3 M guanidine hydrochloride at 25 °C for 30 min. The reduced BSA was subsequently succinated by reacting it with 4 mL of a 20 mM maleic anhydride solution in diethyl ether at 25 °C for 1 h. The succinated BSA was dialyzed using a Slide-A-Lyzer dialysis cassette (3500 MWCO; Thermo Scientific, Rockford, IL, USA) before use in enzyme-linked immunosorbent assay (ELISA).

### 3.5. ELISA

ELISA was performed as previously described [19]. Briefly, 96-well immunoplates (Thermo Scientific) were coated with 0.1 mL of a 1 μg/mL succinated BSA solution in PBS and incubated overnight at 4 °C. The wells were washed three times with PBS containing 0.05% Tween 20 (PBS-T) and blocked with 0.5% gelatin hydrolysate in PBS for 1 h at room temperature. Following blocking, the wells were washed three times and incubated for 1 h at room temperature with 0.1 mL of a 2SC monoclonal antibody solution (1 μg/mL in PBS-T) [20]. After washing three times, wells were incubated with 0.1 mL of a 1:5000 dilution of HRP-conjugated goat anti-mouse IgG antibody (KPL, Gaithersburg, MD, USA) in PBS-T for 1 h at room temperature. After three additional washes, wells were treated with *O*-phenylenediamine dihydrochloride (OPD; Fuji Film Wako Pure Chemical, Osaka, Japan) for 5 min. The reaction was stopped by adding 0.1 mL of 1.0 M sulfuric acid, and absorbance was measured at 492 nm using an Infinite 200 PRO M Plex (TECAN, Männedorf, Switzerland).

### 3.6. Peptide Synthesis

Peptides were synthesized using a solid-phase method with Fmoc chemistry. Fmoc-amino acids were activated with HOBt, HBTU, and DIEA in NMP. Deprotection of the Fmoc group was achieved with 20% piperidine in NMP. During coupling cycles, Fmoc-amino acids were added in a 10-fold molar excess. The *N*-terminus was acetylated using acetic anhydride in the presence of DIEA. Final deprotection and peptide cleavage from the resin were performed using a cleavage solution containing 95% TFA, 2.5% triisopropylsilane, and 2.5% H_2_O for 3 h at room temperature. Peptides were purified using HPLC on a C18 column (9.4 × 250 mm) with a mobile phase of 0.05% TFA in H_2_O/CH_3_CN. Analytical HPLC confirmed peptide purity at >95%, and peptide masses were verified by LC-QTOF-MS.

### 3.7. Statistical Analysis

All experiments were performed with at least three independent replicates. Data are presented as the mean ± standard deviation.

## 4. Conclusions

To understand the role of PTMs of proteins in vivo, it is essential to employ chemical synthesis methods—at least on a laboratory scale—for creating amino acids, amino acid derivatives, peptides, and proteins with these modifications. Such compounds are crucial for generating specific antibodies that recognize target modifications and for investigating protein–protein interactions in detail [21,22,23,24,25,26,27]. Succination (2SC), which occurs through the reaction of accumulated fumaric acid with Cys residues in proteins, has been implicated in the onset and progression of various diseases, including lifestyle-related disorders [6,8,28]. Recent studies have revealed a 2SC degradation pathway in *Bacillus subtilis* involving three enzymatic reactions [29], highlighting a novel aspect of non-enzymatic PTM metabolism. This discovery underscores the importance of investigating whether similar metabolic pathways exist in mammals, given the potential contribution of succination to dysfunction of mitochondria-related diseases.

In this study, we introduced a new synthetic method for producing succinated compounds. Using maleic anhydride, succination of amino acids, peptides, and proteins can be performed more efficiently, rapidly, and under milder conditions than conventional methods. Additionally, this method is cost-effective and does not require specialized equipment for HCl hydrolysis, making it highly accessible. The synthesis of 2SC peptides or proteins with maleic anhydride generally necessitates the protection of free amino groups. As demonstrated, the use of Boc group protection followed by deprotection post-succination offers an effective approach. The reaction using maleic anhydride is useful for the chemical synthesis of succinated compounds; however, since maleic anhydride rapidly hydrolyzes in aqueous solutions, it is not currently applicable to in vivo succination studies. As shown in Figure S1b, succination using maleic acid results in lower reaction efficiency and produces by-products (dimerization of thiol compounds) compared to maleic anhydride. However, since maleic acid is stable in aqueous solutions, it is considered useful for cultured cell experiments. In conclusion, the simple and efficient chemical synthetic method developed in this study provides a valuable tool for future research into succination.

## Figures and Tables

**Table 1:** List of peptides identified by MS through the succination reaction using model peptides.

Peptide No.	Sequence ^a^	Theoretical Mass of the Unmodified Peptide ^b^ (*m*/*z*)	Observed Mass ^b^ (*m*/*z*)
1	Ac-C(Ahx)2K(Bio)Y	510.7663 (+2)	568.7720 (+2)
2	HHHHHHGYC	582.7363 (+2)	689.7424 (+2)
3	STDY	485.1878 (+1)	485.1875 (+1), 583.1865 (+1)
4	INSRW	675.3573 (+1)	675.3562 (+1), 773.3565 (+1)
5	KRHGMDGY	482.2269 (+2)	482.2274 (+2), 531.2278 (+2), 580.2286 (+2)

^a^ All amino acids are represented by single-letter codes, except for special amino acids. Ahx, aminohexanoic acid; K(Bio), *N*-ε-(biotinylcaproyl)-lysine. ^b^ The charge of the predicted or observed mass is indicated by a numerical value in parentheses.

## Data Availability

Data are contained within the article.

## Supplementary Materials

The following supporting information can be downloaded at: https://www.mdpi.com/article/10.3390/molecules30030571/s1, Figure S1: HPLC profile of the reaction products of Ac-Cys and dicarboxylic acid; Figure S2: The reaction mechanism of Ac-2SC and by-product formation; Figure S3: HPLC profile of the reaction products of Cys and maleic anhydride; Figure S4: Recognition of the prepared 2SC-BSA by anti-2SC monoclonal antibodies.
