# Supplementary material for: Rapid and Efficient Synthesis of Succinated Thiol Compounds via Maleic Anhydride Derivatization
Source: Molecules. 2025 Jan 27;30(3):571. doi: 10.3390/molecules30030571 (PMC11820211; doi:10.3390/molecules30030571)
Supplement: Supplementary file 1 [file molecules-30-00571-s001.zip › molecules-3426629-supplementary.pdf]

Supplementary Materials

Rapid and Efficient Synthesis of Succinated Thiol Compounds *via* Maleic Anhydride Derivatization

Hiroshi Yamaguchi [1,2*], Hikari Sugawa [1], Himeno Takahashi [2], and Ryoji Nagai [1,2]

[1] Department of Food and Life Science, School of Agriculture, Tokai University, 871-12 Sugido, Mashiki, Kamimashiki, Kumamoto 861-2205, Japan

[2] Graduate School of Bioscience, Tokai University, 871-12 Sugido, Mashiki, Kamimashiki, Kumamoto 861-2205, Japan

*Correspondence: yamahiro@tokai.ac.jp; Tel.: +81-96-386-2661

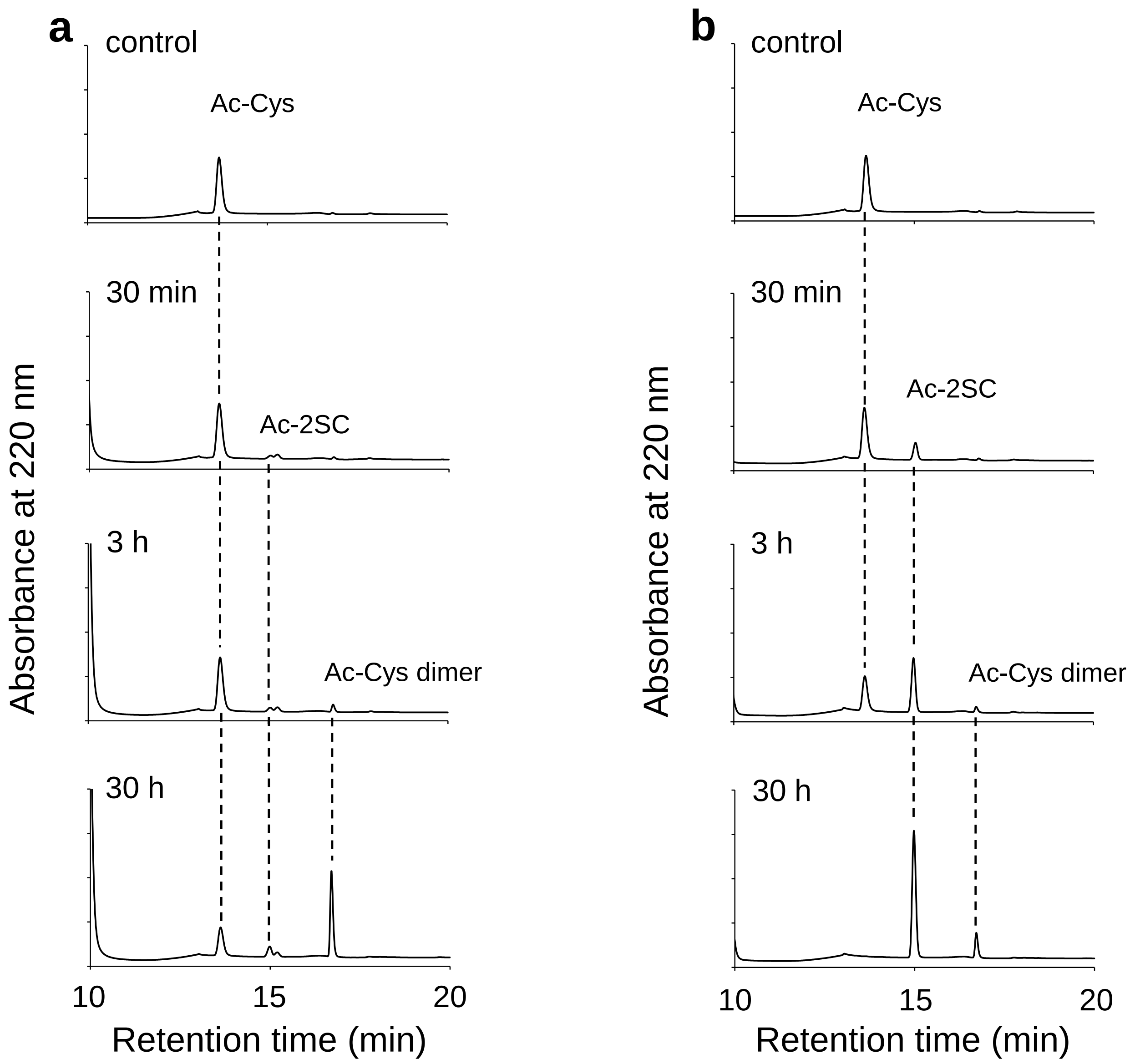

Figure S1. HPLC profile of the reaction products of Ac-Cys and dicarboxylic acid. **(a)** Fumaric acid. **(b)** Maleic acid. The succination reaction was carried out at 25°C in 100 mM phosphate buffer (pH 7.0). The effects of reaction time on succination were evaluated. The concentrations of Ac-Cys and dicarboxylic acid were 0.2 μmol and 4 μmol, respectively.

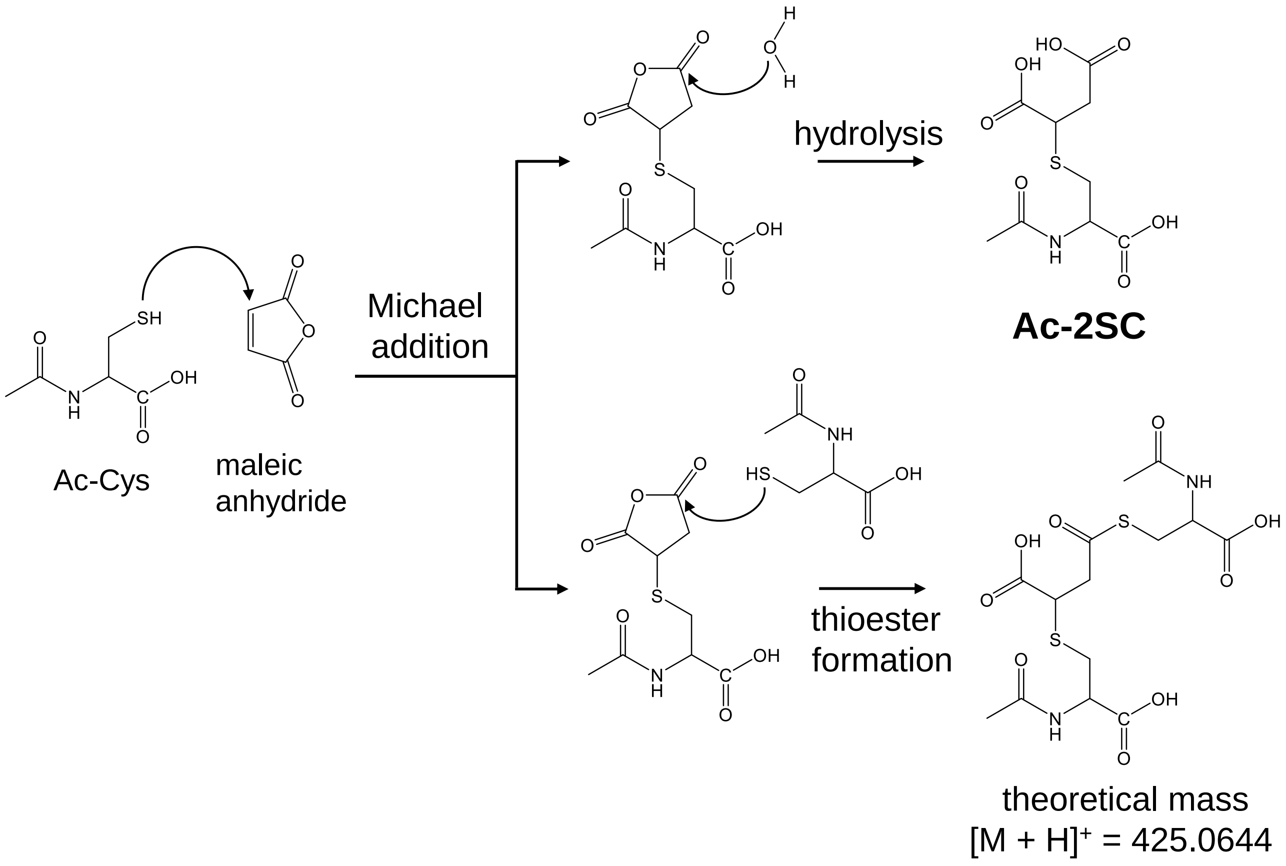

Figure S2. The reaction mechanism of Ac-2SC and by-product formation.

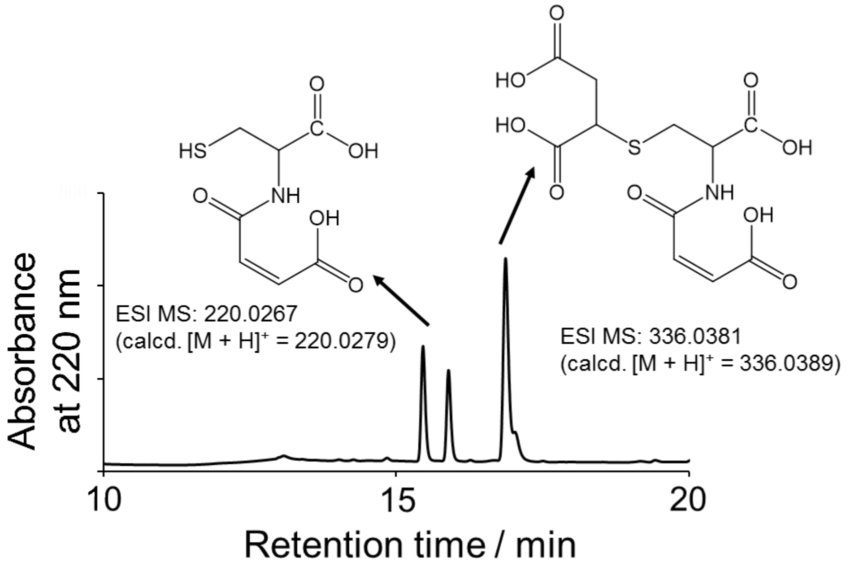

Figure S3. HPLC profile of the reaction products of Cys and maleic anhydride. The succination reaction was carried out at 25 °C for 30 min in 100 mM phosphate buffer (pH 7.0). The concentrations of Cys and maleic anhydride were 0.2 μmol and 4 μmol, respectively.

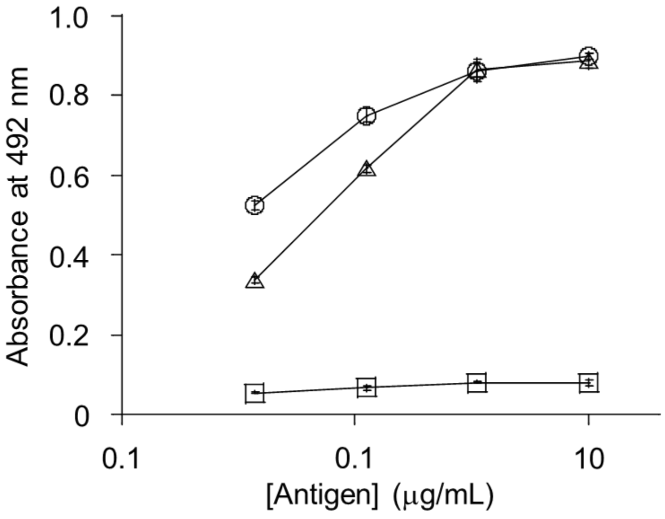

Figure S4. Recognition of the prepared succinated BSA by anti-2SC monoclonal antibodies. BSA modified with maleic anhydride (open circle), BSA modified with maleic acid (open triangle), and BSA (open triangle). The graph shows the mean ± standard deviation for at least three experiments.
